# Two hits are better than one: rational dual strategy efficiently fights neuroblastoma

**DOI:** 10.1038/s41392-024-01827-y

**Published:** 2024-04-30

**Authors:** Jacques Zimmer, Camille Rolin, Markus Ollert

**Affiliations:** 1 Department of Infection and Immunity, Luxembourg Institute of Health, Esch-sur-Alzette, Luxembourg; 2 University of Luxembourg, Esch-sur-Alzette, Luxembourg; 3 Department of Dermatology and Allergy Center, Odense Research Center for Anaphylaxis (ORCA), University of Southern Denmark, Odense, Denmark

**Keywords:** Molecular medicine, Paediatric cancer

The recently published landmark paper by Bergaggio et al. from Roberto Chiarle’s group in *Cancer Cell*^1^ investigates an elegant double-hit strategy to improve anti-cancer immunotherapy. This important approach demonstrates in a preclinical model that chimeric antigen receptor (CAR)-T cell treatment can be rendered more efficient by increasing the cell surface expression of the CAR target structure via a small molecule.

The precise topic of the study, published together with a “Preview” by Marco Ruella,^2^ focuses on neuroblastoma. This tumor consists in a malignant proliferation of neuroblasts and can occur at different extracranial sites of the organism. It preferentially appears in children, where it is one of the most frequent cancers, and young adults, and has a severe prognosis in high-risk cases. Fortunately, newer treatment approaches, such as immunotherapy or anaplastic lymphoma kinase (ALK) inhibitors, can be successfully applied to improve the long-term outcome, at least in a substantial number of patients.^3^

The authors^1^ demonstrate in this manuscript in mice that CAR-T cells targeting the ALK, frequently highly expressed on the surface of neuroblastoma cells, efficiently eliminate the tumor cells. In contrast, efficacy is much lower in case of tumors that express low levels of ALK, which is fully coherent with observations that CAR-T cell approaches need a high antigen density on the targets to reach a maximal effect.^4^ However, this obstacle can be removed through the administration of the ALK inhibitor lorlatinib, which not only reduces tumor growth on its own but also increases surface expression of ALK on the neoplastic cells and thus renders them likewise highly susceptible to the CAR-T cells.

**Fig. 1:**
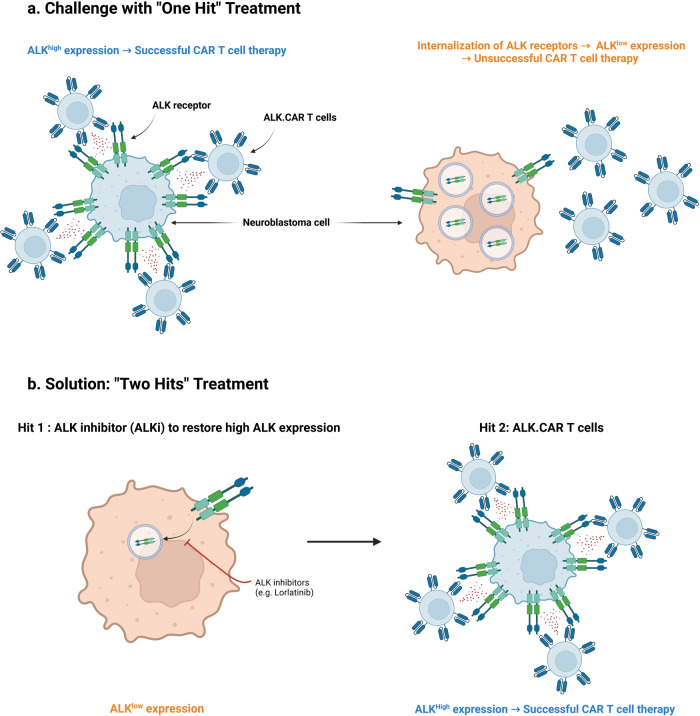
The “double-hit” approach for the treatment of neuroblastoma. **a** Anaplastic lymphoma kinase (ALK) is an antigen frequently expressed at high levels on the surface of neuroblastoma cells. CAR-T cell therapy targeting ALK (ALK.CAR-T cells) demonstrate significant efficacy when this antigen is abundantly present. However, upon activation, ALK receptors are internalized and degraded, decreasing therefore their surface expression and altogether the efficacy of ALK.CAR-T cells. **b** The integration to the therapeutic strategy of ALK inhibitors (ALKi), capable of restoring high ALK surface expression, provides an opportunity to overcome this limitation. Consequently, the synergistic use of ALK inhibitors alongside ALK.CAR-T cell therapy emerges as a promising strategy to address the issue of antigenic variability in neuroblastoma cells. The figure was created with BioRender.com

This “double-hit” strategy was established in in vivo mouse models of metastatic neuroblastoma with high and low ALK expression, respectively. The ALK.CAR-T cells were also cytotoxic to human neuroblastoma cell lines and primary tumor samples in vitro. They were tested in parallel with CD19.CAR-T cells (negative control, the CD19 antigen being a B lymphocyte marker not present on neuroblastoma cells) and, as a positive control, CAR-T cells against the validated and clinically targetable neuroblastoma antigen GD2. Similar GD2.CAR-T cells had already been successfully evaluated in clinical trials.^5^ Whatever the model system, the results were always the same and demonstrated a significant activity of ALK.CAR-T cells against high-ALK neuroblastoma and against low-ALK neuroblastoma, if combined with lorlatinib in the latter case (Fig. 1). However, small metastatic tumors with minor levels of ALK expression, probably too low to be detected by the CAR-T cells, remained in the liver even after the combination treatment. The underlying mechanisms of the variable ALK surface expression are not yet completely elucidated, although partly dependent on the mutational status of this oncogenic driver.^1^

It is widely accepted among scientists and clinicians working in the cancer field that a lot of progress is still needed to overcome the hurdles to constantly effective and durable responses to tumor therapy, such as surgery, radiotherapy, chemotherapy, and, the major groundbreaking hope, immunotherapy. These approaches can be used alone or, to increase the likelihood of a positive response, in combination. Many clinical trials evaluating various aspects of immunotherapy are currently ongoing, and usually need a pre-validation step in animal models, as in the example discussed here.^1^

The authors^1^ checked for ALK expression in normal tissues and found it only in enteric neurons, whereas other studies described ALK in some types of brain neurons, glial cells and endothelial cells, worsening concerns about on-target, off-tumor effects. This might be a rationale for future studies in the field, to be sure that healthy tissues are not susceptible to the effects of lorlatinib. In this context, it would also be interesting to perform retrospective analyses of tumor samples before and after lorlatinib treatment, which would allow to check if ALK expression increased. Bergaggio et al. did not detect any toxic effects in the mouse models, even not in intestinal tissues which could theoretically be targeted by the ALK.CAR-T cells. Here, it should be mentioned that the absence of toxicity in mouse models does not necessarily indicate that it will not become apparent in human clinical trials.

More generally, the toxicity and disadvantages of CAR-T cells should not be neglected: they have to be autologous to avoid a graft-versus-host reaction in the recipient, their administration is often accompanied by a severe cytokine release syndrome and neurotoxicity, recently there was even a Food and Drug Administration warning about the potential occurrence of secondary cancers in treated patients, and the costs for the long manufacturing period are high. In contrast, many of these problems could be circumvented through the use of CAR-natural killer (NK) cells with the same high target specificity and, as already published, excellent results in hematopoietic cancers, or the administration of immune cell engagers bridging immune effector and tumor cells.

There is a discrepancy between the rather good efficiency of cellular immunotherapy in hematologic compared to solid tumors. In the latter case, the T cells or NK lymphocytes have to face an immunosuppressive tumor microenvironment that renders them quickly exhausted, excludes them from the penetration into the tumor and actively inhibits them through T regulatory cells, myeloid-derived suppressor cells or cytokines down-modulating their activity and functions, such as transforming growth factor-β.

One of the messages of the Bergaggio paper is that an elegant combination of two treatment options with complementary mechanisms of action can be much more efficient than only one approach alone. Nevertheless, this therapeutic association still remains to be tested in clinical trials in human patients, to optimize the modalities and the precise schedule (parallel or subsequent administration). Clearly and undoubtedly, such a detailed preclinical study than the one discussed here is an optimal argument for progressing to human clinical application in refractory or relapsed neuroblastoma.
